# Application of Chitin/Chitosan and Their Derivatives in the Papermaking Industry

**DOI:** 10.3390/polym10040389

**Published:** 2018-03-27

**Authors:** Zhaoping Song, Guodong Li, Feixiang Guan, Wenxia Liu

**Affiliations:** Key Laboratory of Pulp and Paper Science and Technology (Ministry of Education), Qilu University of Technology (Shandong Academy of Sciences), Jinan 250353, China; lgd@qlu.edu.cn (G.L.); 1043117338@stu.qlu.edu.cn (F.G.); liuwenxia@qlu.edu.cn (W.L.)

**Keywords:** chitosan, papermaking, wet-end, coating, wastewater

## Abstract

Chitin/chitosan and their derivatives have become of great interest as functional materials in many fields within the papermaking industry. They have been employed in papermaking wet-end, paper surface coating, papermaking wastewater treatment, and other sections of the papermaking industry due to their structure and chemical properties. The purpose of this paper is to briefly discuss the application of chitin/chitosan and their derivatives in the papermaking industry. The development of their application in the papermaking area will be reviewed and summarized.

## 1. Introduction

Chitin is the second most abundant natural polymer in the world. The main sources are from two marine crustaceans, shrimp and crabs [1]. Chitin and chitosan are β(1–4) glycans whose chains are formed by 2-acetamide-2-deoxy-d-glucopyranose and 2-amino-2-deoxy-d-glucopyranose units, respectively. Chitosan is generally prepared by the deacetylation of chitin. Having a unique set of biological properties including biocompatibility, biodegradability, and low to absent toxicity [2], chitin and chitosan, as well as their derivatives, have been found to be attractive materials for some high value-added products, including: cosmetics, food additives, drugs carriers, pharmaceutics, and semi-permeable membranes [1,2,3,4].

There are some very detailed review papers on the introduction, processing, and application of chitin and chitosan, addressing the varied application of chitosan in many fields [1,5]. This paper aims to give a short review on the application of chitin/chitosan as well as their derivatives in the papermaking industry. In recent years, studies focused on investigating the applications of chitosan as a papermaking additive, for both internal and surface applications [6], improving the wet and dry strength of paper [7,8], demonstrating the compatibility of chitosan with paper stock components, and its ability to work as a retention and drainage additive [9,10], or as dye fixative in producing coloured paper [11,12]. Meanwhile, the inherent antibacterial properties and the film forming ability of chitosan are also studied for potential applications in papermaking, laying a foundation for fabricating functional papers such as antibacterial paper and greaseproof paper [13,14]. In addition, chitin/chitosan and their derivatives are also used widely as a chelating and coagulating agent for wastewater treatments [15], due to the sorption of dyes, humic acids, metallic ions, bacterial cells, and xenobiotics on chitin/chitosan in wastewater from papermaking and other industries, according to their unique characteristics and properties [16,17,18,19,20]. In this short review, the recent applications of chitin/chitosan and their derivatives in the papermaking industry will be summarized, and the development of their application in the papermaking field will be discussed.

## 2. Wet-End Application

Wet-end is where the slurry of fibers forms a wet paper web on the paper machine. At the wet-end, there is a continuous water phase and a dispersed phase of cellulose fibers. Wet-end chemistry is very complex since there are many components, including cellulose fibers, paper additives, fines, water, etc. Examples of paper additives used at the wet-end are retention aids, strength resins, internal sizing agents, fillers, and so on. The additives are used to achieve specific paper properties and enhance the paper machine efficiency. Due to the complex of the components in papermaking wet-end, the interactions between the cellulose fibers and the additives are very complex. Chitosan can be used in the wet-end of papermaking, for retention and drainage agents, strength agents, or sizing promoters. As for the application of chitosan in wet-end of papermaking, chitosan and their derivatives are first dissolved in water or acid solution, then the solution is added to the pulp suspension before the formation of paper sheets.

### 2.1. As Retention and Drainage Agents

Retention and drainage agents are very important wet-end additives in the papermaking process. They are used to promote the aggregation of fillers, fibers, and other fines in wet-end by electrostatic interactions. Retention and drainage agents are added in the papermaking wet-end to generate flocs by flocculation or coagulation which improves the retention of fillers and fines. If the flocs are formed by coagulation, the addition of retention and drainage aid also impart better drainability to the pulp suspension which allows higher paper machine speed. For retention and drainage agents, they can be natural or synthetic polymers, single- or multi-component systems [21,22,23]. Currently, natural polymers have become more popular than synthetic ones due to increasing environmental awareness [24]. Chitosan and its derivatives appear to be good candidates to be used as retention and drainage agents in papermaking industry and numerous application strategies of chitosan have already been investigated [10,25,26,27,28,29,30,31,32,33]. Nicu et al. compared the ability of three chitosans with different molecular weights to flocculate grounded calcium carbonate (GCC) and pulp/GCC suspensions in papermaking [34]. Chitosans with a higher molecular weight (MW) showed greater flocculation efficiency since it had a stronger affinity towards cellulose fibers [34]. Since the MW and degree of substitution (DS) of quaternary chitosan have a great influence on the properties of chitosan as a retention and drainage aid [34,35], *N*-(2-Hydroxyl-3-trimethylammonio)-propyl chitosan chloride with varying DS and MW were prepared and the effects of the DS and MW of the quaternary chitosan on its adsorption and flocculation properties in alkaline papermaking were studied [28]. Compared with a commercial cationic starch, the results showed that quaternary chitosan had a lower flocculation concentration and a higher flocculation performance, when used to induce the flocculation of CaCO_3_ fillers in alkaline papermaking. It was also found that the absorption of chitosan on CaCO_3_ fillers as well as the flocculation of CaCO_3_ dispersion were significantly improved by increasing the DS of quaternary chitosan from 43% to 93% due to the enhanced electrostatic interactions between quaternary chitosan and negatively charged CaCO_3_ particles [28]. Meanwhile, quaternary chitosan with lower MW demonstrated higher efficiency in inducing the flocculation of CaCO_3_ particles when chitosan with high DS was used. Similarly, Chi et al. [10] reported the retention and drainage-aid behavior of quaternary chitosan(*N*-(2-Hydroxy-3-trimethylammonio)-propyl chitosan chlorider (C-CS)) as retention and drainage-aids for peroxide bleached reed kraft pulp in papermaking system. They found that the drainage rate of pulp suspension was increased significantly upon addition of C-CS, although, there was a small decrease in the mechanical propertiesshen of finished paper due to the improved retention of CaCO_3_ fillers and fibrous fines. The reason for the higher retention rate of quaternary chitosan can be attributed to the electrostatic interaction between the quaternary chitosan and the cellulosic substrates or mineral fillers in the wet-end of a papermaking system [28]. When the chitosan was modified by introducing a quaternary ammonium group, the modified chitosan became soluble in both neutral and alkaline solutions and obtained good mineral binding properties, which is required for anchoring the mineral to the fibers. Furthermore, dual- or multi-component retention systems involving chitosan, such as a chitosan/bentonite particulate retention system, chitosan/silica (SiO_2_) retention system [36,37], and chitosan/cellulose nanofiber (CNF) retention system, have also been used in the papermaking process [38,39]. Quaternary chitosan (QCS)/nano-SiO_2_ retention aid system has been used in flocculation of reed pulp suspension [36]. The results showed that the flocculation was increased with the increasing of SiO_2_ when the fiber substrates surfaces was net positively charged by an adsorbed QCS layer. The effect on the fiber flocculation of electrolyte concentration in the QCS-nano-SiO_2_ system was governed not only by the adsorption of QCS onto the substrate surface but also by the interaction between polyelectrolyte and nano-SiO_2_ particles [36]. Chitosan with a nano-silica retention/drainage system used in recycled cellulosic fibers was studied [37] and the results showed that retention and drainage in recycled waste office pulp was significantly improved in comparison to the control sample. The effect of chitosan/bentonite particulate retention system on the retention and drainage performance of the tobacco pulp, which was used to make tobacco sheets using papermaking technology, was studied [38]. The results showed that the chitosan/bentonite particle retention and drainage system can improve retention and drainage performance of the tobacco slurry significantly [38]. The employment of CNF combined with chitosan as a dual retention system in the papermaking process showed that the introduction of CNF in the presence of chitosan reduced drainage times [39].

### 2.2. As Paper Strength Agents

Strength properties, both dry and wet, are very important to the paper sheets and paper-based packaging materials. However, the hydrophilic nature of cellulose fibers limits the application of paper products, especially for those products used in humid surroundings that require high strength properties. There are two kinds of paper strength agents, including synthetic polymers and natural renewable polymers, used most frequently in the papermaking industry. Among the bio-based renewable polymers, chitosan with important functional groups such as hydroxyl, amino, and even acetamido groups has been found to be effective as a dry strength agent in the papermaking industry [28,37,40,41,42,43,44,45,46,47,48]. The structure of chitosan is similar as cellulose, making it possible to have strong bonding with fibers thus giving a dry and wet strength in papermaking. It was also observed that the amino groups on chitosan could react with cellulose’s aldehydes and subsequently produce covalent bonds [49].

Chitosan can be used alone as a strength agent in papermaking. For example, the effect of chitosan on properties of handsheets made from bleached eucalypt pulp has been quantified [50], and the research concluded that chitosan has the potential to be used as a dry strength additive in neutral, acidic, or alkaline conditions depending on process requirements. Meanwhile, the effects of shrimp chitosan on the physical properties of handsheets were investigated by Khantayanuwong et al. [51]. The results showed that most of the mechanical properties of shrimp-chitosan-treated handsheets such as the bursting index, folding endurance, tensile index, modulus of elasticity, and tensile energy absorption were greatly increased with the addition of chitosan at 0.25–0.5 o.d. wt. % of pulp, except that there was no change in the tearing strength.

In order to achieve satisfying strength properties of paper sheets, chitosan is often used in combination with other products. For example, synergy of carboxymethyl cellulose (CMC) and modified chitosan [52], and synthesized chitosan-complexed starch nanoparticles [53] have been adopted to enhance the strength properties of a cellulosic fiber network. The combination of chitosan with bentonite microparticles to act as a wet-end additive system for paper reinforcement has been studied [54]. It was suggested that the bentonite may make a bridging between different chitosan molecules, making them act like a higher-mass cationic polymer and chitosan showed potential as a dry strength additive in mixed hardwood chemical-mechanical pulp in acidic pH. Additionally, chitosan was used in combination with cationic starch as dry-strength agents to improve the strength properties of bagasse paper [55]. The results showed that the dry-strength agent acted as a protecting film or glazed on the surfaces of bagasse paper handsheets, which had a positive impact on the pulp properties. This work showed the feasibility of using chitosan and cationic starch as dry-strength additives for application of non-wood materials in the paper industry. In another study, chitosan, cationic starch and poly vinyl alcohol (PVA) were used in various sequences to find out the optimal combination for improving both wet and dry tensile strength of old corrugated containerboard (OCC) furnish and the best results in wet and dry tensile strengths were simultaneously achieved using sequential PVA-chitosan-cationic starch [56]. Moreover, the synthesis and application of chitosan-complexed starch nanoparticles for improving the physical properties of recycled paper furnish (OCC), was also studied [53] and provided a uniquely renewable and useful approach to enhance the mechanical properties of pulp while maintaining environmental compatibility, industrial compatibility, and paper qualities. Other complexes such as xylan/chitosan complex [44], CMC/chitosan complex [57], maleic anhydride-acylated chitosan [58], and soy flour combined chitosan dual system complexes [59] have been prepared and used to enhance paper strength. The results indicated that those complexes are potential strength agents used in the paper industry. Soy protein flour−DTPA (Diethylenetriaminepentaacetic acid)–chitosan agent [60] and nanocellulose-DTPA-chitosan agent [61] were prepared by Salam et al. and the performance of those complexes used as dry-strength agents in papermaking was investigated. The results revealed that those agents provided increased tensile and burst strength for the modified OCC pulp sheet and significantly increased gloss and water repellency with diminished surface roughness.

Chitosan and its derivatives have been identified as the potential dry and wet strengthening additive for papermaking. The potential advantages can be illustrated in the film forming property of chitosan improves the surface properties of paper [62], the formation of hydrogen bonds [63] and the imine [64]. The paper strength is a function of fiber-fiber bond strength, fiber strength, and sheet formation. However, there are always air voids between the fibers in fiber networks after sheet formation. In recent years, research found that fiber-fiber hydrogen bonding can be greatly enhanced by the use of strength additives. When chitosan is added to the wet-end of papermaking, a film covering the fiber crossing areas could lead to stronger bonds by welding the surfaces together. Meanwhile, the hydroxyl groups of chitosan could form hydrogen bonds with weakly polar areas of fiber surfaces, therefore contributing to paper strength development if the fibers come sufficiently close in order to meet the required geometry conditions. Therefore, the film-forming potential of chitosan not only facilitates the formation of van der Waals forces between the fibers but also provides suitable conditions for hydrogen bonds to occur [63]. Moreover, the formation of imine has been proposed as an important contribution to the ability of chitosan to increase wet strength [64] and studies into the nature of chitosan promoted discoloration of paper have proposed and provided evidence of imine formation [65].

### 2.3. As ASA Sizing Promoter

Chitosan has also been reported to act as a paper sizing promoter to improve the alkenyl succinic anhydride (ASA) emulsion stability. Liu’s group reported the capability of chitosan promoting the sizing performance of ASA emulsion stabilized by montmorillonite [66] or laponite [67] due to its large amount of amino groups. In this role, chitosan, with low molecular weight, could significantly improve the sizing performance of ASA emulsion without inducing the flocculation of the ASA droplets at low charge amounts.

### 2.4. As the Other Wet-End Additives

In addition to the strength properties, chitosan and some derivatives can bring about other properties to paper sheets such as electrical properties [68], antibacterial properties [69,70], and barrier properties [71]. Work done by Nada et al. [68] focused on studying the dependence of paper sheet strength properties on the composition of additives—chitosan and its derivatives, which enhanced the strength properties and the dielectric properties of unaged and aged paper sheets. Research on the synergistic effects of chitosan–guanidine complexes used as functional additives for paper on enhancing wet-strength and antimicrobial activity of paper was carried out, and the chitosan-guanidine complexes synergistically improved wet-strength and antimicrobial activities [69]. Similarly, the chitosan-cellulose blends provided mechanical, antibacterial and water barrier properties [70]. Another study on the pulp-fiber-chitosan sheets investigated the effects of incorporating chitosan or chitosan-acetic acid salt as oxygen-barrier or air-barrier components on the packaging-related mechanical and barrier properties. The results showed that the addition of the chitosan solution to the pulp slurry led to a substantial loss of fiber and chitosan through the wire screen and consequently a low grammage and high sheet porosity and air permeance [71].

## 3. Surface Coating Application

As a linear carbohydrate biopolymer, chitosan has a very similar chemical structure to cellulose. It is easily absorbed onto the cellulosic surface of fibers due to its chemical affinity. Chitosan has been applied widely to improve some properties of cellulose-based materials, especially those of cellulose fibers and paper sheets [72]. Chitosan coatings on the surface of cellulose fiber network or paper sheets have been considered for antimicrobial [73,74] or antibacterial purpose [75,76], as well as enhancing water vapor barrier properties [77], oxygen barrier properties, grease barrier properties [14,77,78,79], anti-electrostatic effects, dyeability promoter of paperboard [80], and increasing the mechanical strength [64,81,82] or the surface property of paper products [83]. As for the surface coating application, chitosan can be used in the form of aqueous solution [76] or emulsion [84], via rod coater [14], bar coater [76], wire bar coater [84], multicoated [85] or size press [79], to be transferred onto the paper surface to endue paper with specific characteristics.

### 3.1. Antibacterial and Antimicrobial Properties

Antibacterial paper is highly significant to living environments and health condition, while also being widely used as food wrappers, hospital paper, indoor environmental protection paper, and sanitary paper, etc. [86]. Antibacterial paper can be produced by coating chitosan and its derivatives or chitosan complex systems on the paper surface, since chitosan possesses the antibacterial properties. Chitin and chitosan have been investigated as an antimicrobial material against a wide range of target organisms like algae, bacteria, yeasts, and fungi in experiments involving in vivo and in vitro interactions with chitosan in different forms (solutions, film, and composites), and three possible and accepted antimicrobial mechanisms for chitosan discussed by Goy et al. [87].

Research on antimicrobial properties of chitosan-coated paper by Vartiainen et al. showed that chitosan dissolved in 1.6, 3.2, and 6.4% lactic acid showed antimicrobial activity against *Bacillus subtilis* [73]. Janjic et al. developed biologically active cellulose-chitosan fibers by oxidizing lyocell fibers with potassium periodate followed by a chitosan coating [88]. Chitosan-coated lyocell fibers were prepared by subsequent treatment of oxidized lyocell fibers with a solution of chitosan in aqueous acetic acid. The free amino group of chitosan reacts with an aldehyde to give the corresponding Schiff base with high degrees of substitution. The antibacterial activity of the cellulose-chitosan fibers against different pathogens including *Staphylococcus aureus* and *Escherichia coli* was confirmed in their experiments. Novel antibacterial paper was fabricated by a surface coating based on modified chitosan and organic montmorillonite/Ag nanocomposites complex and the results proved that this study provided basic data for an efficient and safe chitosan-contained antibacterial agent that can be applied in the paper industry [89]. The combination of propolis and chitosan was also used to impart antimicrobial as well as antioxidant capacity to paper and cellulosic packaging materials, improving some fundamental features of paper and food packaging materials [90,91].

### 3.2. Strength and Barrier Properties

Chitosan is selected as the coating material to enhance paper strength and barrier properties due to its good film forming property, and the reactive amino and hydroxyl groups of chitosan have the potential to form hydrogen bonds with fiber surfaces, therefore contributing to paper strength development. Wang et al. [92] discussed the film formation of chitosan coated on the surface of Kraft paper and they found that there was no chitosan penetration through the Kraft paper, indicating the good film-forming property of chitosan. The resulting paper obtained strongest water vapor barrier properties when there was a higher concentration of chitosan solution at the optimum pH, stirring speed, and those with a thicker coating on the Kraft paper. Gandini [93] reported that the deposition of chitosan films of different thicknesses on uncoated paper sheets not only improved the optical properties of the ensuing surfaces and their printability, but also brought about useful modifications of certain mechanical and permeability properties. In addition, production of coated papers with a water-soluble chitosan derivative was discussed by Fernandes et al. They claimed that paper coated with such chitosan derivative presented superior optical properties, printability, and had better results on aging measurements than the pristine chitosan-coated papers [94]. However, the hydrophobic property of water-soluble chitosan coated paper was limited.

It was said that chitosan can be used as a pre-coating on paper to provide better bonding and a more uniform surface for other processing steps like, e.g., the application of an additional biopolymer layer by extrusion coating [72,95]. This is similar to the layer-by-layer (LBL) assembly technique, which was used to build coating multilayers on the paper surface and employed in improving the properties of paper in the papermaking field. Despond et al. [78] carried out the experiment focusing on the barrier properties of paper coated with chitosan and carnauba wax. Chitosan was first coated to obtain a dense polymer layer at the paper surface, which gave interesting gas barrier properties in the anhydrous state of coated paper. This was followed by the coating of carnauba wax forming a bilayer which led to a hydrophobic surface of the treated paper. Similarly, carboxymethyl cellulose-chitosan complex LBL treatment on cellulose fiber networks was carried out to enhance the wet and dry tensile strength of cellulose fibre networks [57]. Another study showed that the incorporation of sodium alginate in a chitosan formulation significantly improved the fat resistance of the coated paper in comparison with a pure chitosan coating; however, the introduction of cellulose ethers in the chitosan formulations did not improve the fat resistance of coated papers [79]. Zhang et al. [85] used chitosan in combination with beeswax to create a high water vapour barrier property and grease resistance of coated paper. The results showed that as the concentration of chitosan solution increased from 1.0 to 3.0 wt. %, its water vapour transport rate (WVTR) decreased from 171.6 to 52.8 g/m^2^/d but using reduced beeswax coating weight (from 10.1 to 4.9 g/m^2^). It also displayed an enhanced performance of grease resistance. Study on chitosan-caseinate bilayer coatings for paper packaging materials was also reported [96] and the results showed that chitosan significantly increased the elongation at break of coated paper while caseinate led to a decrease in water vapor permeability. Moreover, LBL self-assembly deposition of the chitosan lactate-carboxymethyl cellulose complex previously modified with metal oxide for reinforcement of aged papers was studied [97] and shown to have an excellent improvement in the mechanical properties of the treated paper. Nanofibrillated cellulose-chitosan nanocomposite films were prepared and used for paper coating [98]. The coating of nanocomposite films improved the tensile strength properties and grease-proof properties of the coated paper while decreasing the porosity and water absorption of paper. However, the water vapor permeability was not affected.

### 3.3. Other Coating Applications

In addition to the applications of chitosan coating on paper to create oxygen gas barriers, water vapor barriers, and grease or fat barriers of coated paper, another interesting topic should be the preparation of some intelligent chitosan coated paper based materials [6,99,100]. For example, an interesting study showed that an intelligent and biodegradable temperature indicator packaging material could be developed through incorporating a heat-sensitive pigment (anthocyanin-ATH) into chitosan-acetic acid dispersion that was applied as surface coating on card paper. This smart packaging materials can indicate temperature variations in a specific range by irreversible visual colour changes [100]. Chitosan as a paperboard coating additive for use in HVAC (heating, ventilation, and air conditioning) applications has been reported [6]. Commercial chitosan and fungal chitosan solution were coated onto unbleached Kraft paper to be an alternative to phenolic resin coatings and the results indicated the potential of chitosan coated paper for manufacturing evaporative cooling pads used in livestock enclosures.

## 4. Wastewater Treatment

A variety of pollutants are generated from pulp and papermaking mills depending on which process is used. The high amount of water and various chemicals used in the complicated processes in papermaking industry generates large amounts of contaminated wastewater. The pulp and paper industry is considered as a big polluter in the world. Therefore, learning how to deal with the papermaking wastewater is a big issue. Advanced wastewater treatment technologies are mandatory to reduce fresh water consumption with minimum detrimental effects on papermaking operations and paper quality [101]. Pokhrel et al. listed considerable methods for dealing with the wastewater in the papermaking industry, including physicochemical, biological, fungal, and integrated treatment processes. Chitosan was mentioned in the coagulation and flocculation method, which is normally employed in the tertiary treatment in the case of pulp and paper mill wastewater treatment [17].

Coagulation and flocculation is an important secondary treatment procedure in the removal of turbidity, colloids, and natural organic matter during water treatment processes [102]. Chitosan has the characteristics of both a coagulant and a flocculant with high cationic charge density, long polymer chains, and acts as a bridge for aggregates and precipitation. It may be considered as one of the most promising bioflocculants for environmental and purification purposes [16,103]. Many studies showed that chitosan-based flocculants have many advantages, including their widespread availability, environmental friendliness, biodegradability, and prominent structural features when they are used in the wastewater treatment [104]. Wang et al. [105] reported a novel cationic chitosan-based flocculant with a high water-solubility for pulp mill wastewater treatment. The flocculate was synthesized through grafting (2-methacryloyloxyethyl) trimethyl ammonium chloride (DMC) onto chitosan initiated by potassium persulphate. The results showed that this chitosan-based flocculant had an excellent flocculation capacity and its flocculation efficiency was greater than that of polyacrylamide. In another study, chitosan dissolved in acetic acid and was used as a flocculating agent in the flocculation of cardboard industry wastewater treated by a biological process in an aerated lagoon [16]. Compared with commercial grade polyaluminium chloride (PAC), an extensively used flocculant in wastewater treatment, chitosan induced a more efficient flocculating process. Chitosan lowered the chemical oxygen demand (COD) by over 80% and turbidity by more than 85% which were much higher than PAC did. Moreover, using chitosan as flocculant generated bigger flocs makes settling faster than in the case of using PAC. Meanwhile, chitosan-induced flocculation removed more residual colour and led to a significant decrease in the amount of heavy metals present in the effluent.

In order to investigate the effects of molecular weights on the chitosan performance, Miranda et al. evaluated two native chitosans with different molecular weights on a laboratory scale for their effectiveness in the removal of contaminants from papermaking process waters by dissolved air flotation [106]. The use of chitosan quaternary derivatives and the use of the native chitosans in combination with anionic bentonite microparticles have also been tested. The results demonstrated a high efficiency of the native chitosan products at intermediate dosages and their efficiency was enhanced by the combined addition of bentonite. Quaternary derivatives obtained lower efficiency than the base chitosan used. The main reason for this was the lower charge density of the quaternary derivatives compared to the native chitosans at the operational conditions.

Many studies proved that chitosan can also be used in combination with other polymers for wastewater treatment. Tong et al. [107] and Ganjidoust et al. [108] carried out a comparative study of horseradish peroxidase (chitosan) and other synthetic polymers, including hexamethylene diamine epichlorohydrin polycondensate (HE), polyethylenimine(PEI), and polyacrylamide(PAM), to remove lignin and other kinds of chlorinated organic compounds from pulp and paper industrial wastewater. The results showed that modified chitosan was far more effective in removing these pollutants than other coagulants. Zeng et al. [109] prepared a composite flocculant that consisted of chitosan, polymerized ferrous sulfate (PFS), and PAM to treat papermaking wastewater. This composite flocculant has economic and environmental benefits due to its lower price and higher efficiency. Liu et al. reported a macroporous resin with a methyl acrylate matrix and coated with chitosan of various molecular weights through glutaraldehyde crosslinking for the treatment of whitewater from papermaking after pectinase and lipase were immobilized on the resin coated with chitosan [110]. The macroporous resin, immobilized with dual-enzymes, was proved useful for the treatment of whitewater in the papermaking industry by reducing the cationic demand and pitch deposits in whitewater by 58% and 74%, respectively. Another study reported the preparation of cross-linked chitosan beads with immobilized pectinase, which were used to investigate the effects of enzymes in lowering pectins or polygalacturonic acids (PGA) concentration in papermaking industries. The results showed that the PGA-absorption capability of chitosan beads was greatly affected by its cross-linking degree [19]. This revealed the potential for cross-linked chitosan beads which lowers canionic demand of PGA by solute adsorption and pectinase immobilization for potential use in water treatment of the papermaking industry. Petzold et al. studied the removal of dissolved and colloidal substances (DCS) in paper cycling water with modified starch and chitosan compared with a control [111]. Results revealed that turbidity and total organic carbon (TOC) were lowered especially due to charge interaction, whereas the increase in surface tension is mainly caused by the hydrophobic character of the modified natural polymers.

## 5. Other Applications

Other applications of chitosan in the papermaking or papermaking-related industries have also been studied, such as modification of cellulose fibers [65,112], blending with cellulose to prepare chitosan/cellulose blend beads [113], crosslinking with cellulose nanofibers to form nanopaper with water-resistant and transparent properties [114], etc. As such, photochromic paper from wood pulp modified via LBL assembly chitosan-spiropyran on pulp fibers has been studied [112]. The LBL–treated fibers were compatible with pulp fibers, which gave a highly effective method to impart the photochromic characteristic to paper. Chitosan with two different molecular weights were employed as flocculant to recover the dissolved lignocellulosic materials of industrially produced pre-hydrolysis liquor (PHL). The addition of chitosan causes the precipitation of dissolved lignocellulosic materials. Chitosan with higher molecular weight induces more precipitation of dissolved lignocellulosic materials at a lower concentration [115].

## 6. Conclusions

Chitin and chitosan and their derivatives have a wide range of applications in the papermaking industry. They can be employed to solve numerous problems in wet-end chemistry and wastewater treatment such as improving the efficiency of the paper machine, enhancing paper strength, or to prepare antibacterial, high barrier, intelligent paper-based packaging materials. Together, with the biodegradable nature of chitosan, it appears that chitosan can be an interesting and promising candidate for environmentally-friendly high value-added paper production. Up to now, the economy of the chitosan application in large scale in the papermaking industry has not been considered.
